# The Learning Objective Catalogue for Patient Safety in Undergraduate Medical Education – A Position Statement of the Committee for Patient Safety and Error Management of the German Association for Medical Education

**DOI:** 10.3205/zma001009

**Published:** 2016-02-15

**Authors:** Jan Kiesewetter, Johanna Gutmann, Sabine Drossard, David Gurrea Salas, Wolfgang Prodinger, Fiona Mc Dermott, Bert Urban, Sven Staender, Heiko Baschnegger, Gordon Hoffmann, Grit Hübsch, Christoph Scholz, Anke Meier, Mirko Wegscheider, Nicolas Hoffmann, Theda Ohlenbusch-Harke, Stephanie Keil, Christian Schirlo, Lisa Kühne-Eversmann, Nicole Heitzmann, Alexandra Busemann, Ansgar Koechel, Tanja Manser, Lena Welbergen, Isabel Kiesewetter

**Affiliations:** 1 Klinium der Universität München, Institut für Didaktik und Ausbildungsforschung in der Medizin, München, Deutschland; 2 Technische Universität München, Klinikum Rechts der Isar, München, Deutschland; 3 Klinikum Augsburg, Kinderchirurgische Klinik, Augsburg, Deutschland; 4 Klinikum Nordschwarzwald, Calw, Deutschland; 5 Medizinische Universität Innsbruck, Sektion für Hygiene und medizinische Mikrobiologie, Innsbruck, Österreich; 6 Universitätsklinikum Bonn, Institut für Patientensicherheit, Bonn, Deutschland; 7 Klinikum der Universität München, Institut für Notfallmedizin und Medizinmanagement (INM), München, Deutschland; 8 Spital Männedorf, Institut für Anästhesie und Intensivmedizin, Männedorf, Schweiz; 9 Klinikum der Universität München, Klinik für Anästhesiologie, München, Deutschland; 10 Technische Universität Dresden, Medizinische Fakultät, Medizinisches Interprofessionelles Trainingszentrum, Dresden, Deutschland; 11 Universitätsklinikum Ulm, Frauenheilkunde und Geburtshilfe, Ulm, Deutschland; 12 Universität Hamburg, Hamburg, Deutschland; 13 Universitätsklinikum Leipzig, Klinik für Neurologie, Leipzig, Deutschland; 14 Universitätsklinikum Hamburg-Eppendorf, Klinik und Poliklinik für Anästhesiologie, Hamburg, Deutschland; 15 Universität Regensburg, Fakultät für Medizin, Zentrum für Lehre, Regensburg, Deutschland; 16 Universität Zürich, Medizinische Fakultät, Dekanat, Zürich, Schweiz; 17 Universität München, Hormon- und Stoffwechselzentrum München, München, Deutschland; 18 Universitätsmedizin Greifswald, Klinik und Poliklinik für Chirurgie, Greifswald, Deutschland; 19 Universitätsklinikum Heidelberg, Klinik für Allgemeine Innere Medizin und Psychosomatik, Heidelberg, Deutschland; 20 Klinikum der Universität München, Kinderklinik und Kinderpoliklinik im Dr. von Haunerschen Kinderspital, München, Deutschland

**Keywords:** Patient Safety, Learning Objective Catalogue, Medical Education

## Abstract

**Background: **Since the report “To err is human” was published by the Institute of Medicine in the year 2000, topics regarding patient safety and error management are in the focal point of interest of science and politics. Despite international attention, a structured and comprehensive medical education regarding these topics remains to be missing.

**Goals: **The Learning Objective Catalogue for Patient Safety described below the Committee for Patient Safety and Error Management of the German Association for Medical Education (GMA) has aimed to establish a common foundation for the structured implementation of patient safety curricula at the medical faculties in German-speaking countries.

**Methods: **The development the Learning Objective Catalogue resulted via the participation of 13 faculties in two committee meetings, two multi-day workshops, and additional judgments of external specialists.

**Results:** The Committee of Patient Safety and Error Management of GMA developed the present Learning Objective Catalogue for Patient Safety in Undergraduate Medical Education, structured in three chapters: *Basics, Recognize Causes as Foundation for Proactive Behavior*, and *Approaches for Solutions*. The learning objectives within the chapters are organized on three levels with a hierarchical organization of the topics. Overall, the Learning Objective Catalogue consists of 38 learning objectives. All learning objectives are referenced with the National Competency-based Catalogue of Learning Objectives for Undergraduate Medical Education.

**Discussion: **The Learning Objective Catalogue for Patient Safety in Undergraduate Medical Education is a product that was developed through collaboration of members from 13 medical faculties. In the German-speaking countries, the Learning Objective Catalogue should advance discussion regarding the topics of patient safety and error management and help develop subsequent educational structures. The Learning Objective Catalogue for Patient Safety can serve as a common ground for an intensified, constructive, subject-specific discussion about these topics at the medical faculties, and guide the implementation of hopefully multiple patient safety curricula in undergraduate medical education.

## Zusammenfassung

**Hintergrund:** Seit dem vom Institute of Medicine im Jahr 2000 veröffentlichten Bericht „To err is human“ rückten die Themen Patientensicherheit und Fehlermanagement zunehmend in den wissenschaftlichen und politischen Fokus. Obwohl sich dem Thema bereits international auf verschiedensten Ebenen angenähert wurde, fehlt bislang eine strukturierte und flächendeckende Ausbildung von Ärztinnen und Ärzten in diesem Bereich.

**Ziele: **Mit dem im Folgenden beschriebenen Lernzielkatalog Patientensicherheit möchte der Ausschuss für Patientensicherheit und Fehlermanagement der Gesellschaft für medizinische Ausbildung (GMA) eine erste gemeinsame Grundlage für die Implementierung strukturierter Patientensicherheitscurricula zur Ausbildung der Medizinstudierenden an medizinischen Fakultäten im deutschsprachigen Raum schaffen.

**Methoden: **Die Erarbeitung des Lernzielkataloges erfolgte unter Beteiligung von 13 medizinischen Fakultäten im Rahmen von zwei Ausschusstreffen, zwei mehrtägigen Workshops und mit Hilfe von Beurteilungen durch externe fachliche Expertinnen und Experten.

**Ergebnisse:** Der vom Ausschuss für Patientensicherheit und Fehlermanagement der GMA entwickelte Lernzielkatalog Patientensicherheit für das Medizinstudium besteht aus den drei Kapiteln: *Grundlagen, Erkennen von Ursachen als Basis für proaktives Handeln und Lösungsansätze*. Die Lernziele innerhalb der Kapitel sind hierarchisch auf drei Ebenen organisiert. Insgesamt beinhaltet der Lernzielkatalog 38 Lernziele. Alle Lernziele wurden mit dem Nationalen Kompetenzbasierten Lernzielkatalog Medizin referenziert.

**Diskussion: **Der vorliegende Lernzielkatalog ist ein Produkt der Zusammenarbeit von Vertretern aus immerhin 13 medizinischen Fakultäten. Er soll im deutschsprachigen Raum die Diskussion des Themas Patientensicherheit und Fehlermanagement im Fach Medizin und die Entwicklung entsprechender Ausbildungsstrukturen voranbringen. Zukünftig kann der Lernzielkatalog Patientensicherheit für das Medizinstudium als Grundlage dienen, um die konstruktive fachliche Auseinandersetzung mit dem Thema an den jeweiligen Fakultäten zu vertiefen und die Implementierung möglichst zahlreicher curricularer Strukturen anzustoßen.

## Introduction

Since the report “To err is human” was published by the Institute of Medicine in the year 2000, topics regarding patient safety and error management [1] are in the focal point of interest within science and politics in many countries and in the awareness of the public. In Germany, the number of critical incidents is also not negligible [2]. Substantial aspects of patient safety regulations were tied together in Patientenrechtegesetz, (translation by the author “Laws of Patients’ Rights”), which strengthened the position of the patients [http://www.bgbl.de/xaver/bgbl/start.xav?startbk=Bundesanzeiger_BGBl&jumpTo=bgbl113s0277.pdf cited 6-23-2015]. In particular, the procedure after potential errors has been legally regulated. While this this has been an important step in the right direction on the legislative part, it does not tackle the roots of the problem. Despite some positive developments in diverse specialties and areas (i.e. the establishment of the first Institute for Patient Safety at a German university, which dedicates its focus on this topic), no consistent rethinking in medicine has taken place until today, 10 years after the report of the IOM [3], [4].

Requests to integrate the topic ‘patient safety’ in medical education have been existing for a while. For example, the World Health Organization (WHO) has published a guideline how to develop medical curricula for the topic of patient safety („Patient safety curriculum guide“ [5]). The European Union has passed similar recommendations and has explicitly suggested to teach patient safety topics in the education of all medical professions [http://www.eu-patient.eu/globalassets/projects/eunetpas/guidelines_final_22-06-2010.pdf cited 6-23-2015]. As early as 2007, the Swiss Academy of Medical Sciences has published recommendations for the education in patient safety and error culture in undergraduate and graduate medical education [6]. Regarding the professional societies, the European Society for Anaesthesiology has targeted graduate medical education courses in patient safety through their European Patient Safety Course since 2007 [https://www.esahq.org/patient-safety/patient-safety/patient-safety-and-quality-committee cited 9-30-2015]. In Germany, the establishment of the German Coalition for Patient Safety in 2005 and, especially, with the formulation of the learning goal catalogue „Wege zur Patientensicherheit“ (translation by the author “Road to Patient Safety”) directed to education of all healthcare professions, efforts have been taken to integrate the topic in educational structures [7]. 

At the foundation of this system – the education of physicians – a structured and comprehensive medical education is thus far missing; topics of development, prevention and handling of critical incidents are only covered rudimentarily. And this is the case even though physicians will be confronted with critical incidents during their career, which appear following personal errors, teamwork errors, or errors in the system. 

As the National Competency-based Catalogue of Learning Objectives for Undergraduate Medical Education (NCLM) [8] has been passed, the chance appears to take concrete steps towards integration of this topic in the education of future physicians. From the perspective of the Committee of Patient Safety and Error Management of the GAME, the primary objective of the educational structure is to sensitize the young medical professions for the topics and anchor a self-concept for the role of a physician with regard to medical error culture. This professional self-concept should be focused to react to errors not with individual-related blame (“culture of blame”) but instead towards an open and constructive culture of communication and exercise effective error management. This error management should enable the discussion of errors with patients and relatives as well as to speak openly and neutrally within the team so that chains of error development can be analyzed and prevention strategies can be established. This cultural change towards a vivid safety culture needs to be integrated.

### Goals

The Learning Objective Catalogue for Patient Safety described below the Committee for Patient Safety and Error Management of the German Association for Medical Education (GMA) has aimed to establish a common foundation for the structured implementation of patient safety curricula at the medical faculties in German-speaking countries. The Learning Objective Catalogue is supposed to be easily manageable for those involved in the structuring and implementation of medical education. By referencing the NCLM, it is possible to synergistically integrate the topic of patient safety with other curricular innovations.

## Methods

The foundation for this position statement was set at the Annual Meeting of the German Association for Medical Education in Graz, Austria with the establishment of the Committee for Patient Safety and Error Management. The goals and workflow were coordinated by 15 members from 8 medical faculties. In march 2014, the first committee meeting took place.

To target the goal to develop evidence-based learning objectives, the members utilized the learning catalogues that were published to that date [5], [9], [10]. The publications were discussed at length and their applicability for curricular innovations in German undergraduate medical education was verified. Additionally, an example of a curricular implementation from Switzerland was presented [11]. The material offered an elaborate, extensive, and multi-professional perspective. However, the requirement to pose concrete guidelines for German medical faculties to establish respective curricula was not met due to the extensive approaches taken. The existing learning objective catalogues were taken as a sound foundation for structural mental guidance to develop a more detailed and more specific learning objective catalogue for patient safety in undergraduate medical education. 

To probe curricular content, top-level categories were composed, partially loosely based on the existing learning objective catalogues. After agreeing on the key aspects and their order, learning objectives were formulated in two rounds of revision. Learning objectives were allocated to the top-level categories. The first round of revision was set within the committee. For a second round of revision, all learning objectives were sent to 16 professional experts outside the committee to avoid group bias. The revisions suggested by the experts were integrated into the learning objective catalogue and formulations of the learning objectives were harmonized during another committee meeting in March of 2015 in Munich. Eventually, the referencing of the learning objectives with the NCLM was performed by two committee members (FM, WP) 

Overtime, 11 members from 5 additional faculties joined the committee; overall, 26 persons from 13 German-speaking faculties were involved in finalizing this version of the Learning Objective Catalogue. 

## Results

### Structure

The Learning Objective Catalogue is subdivided into three chapters:

1. Basics (ca 10-15%)
2. Recognizing Causes as Foundation for Proactive Behavior (ca 40%)
3. Approaches for Solutions (ca 45-60%)

The percentages are intended to set the scale for the fraction of time and content for the chapter in relation to the entire curriculum.

The order of the individual chapter was worked-out on three levels. Level 1 contains the main categories or topics of the chapter. For some main categories, subtopics are defined on Level 2. Level 3 contains the indexed content of the learning objective. The foci of the individual learning objectives were assigned as the type knowledge (38 learning objectives), and/or the type attitude (15 learning objectives), and/or the type skills (15 learning objectives). 

Tab. 1 shows the complete Learning Objective Catalogue for Patient Safety in Undergraduate Medical Education (see attachment 1 ).

#### In reference with the National Competency-based Catalogue of Learning Objectives for Undergraduate Medical Education

The structure of the Learning Objective Catalogue for Patient Safety in Undergraduate Medical Education is complemented with the three learning goal levels of the NCLM (factual knowledge, procedural and conditional knowledge, and professional competence). The learning objective of the NCLM, which fitted the best to a learning objective of the Learning Catalogue presented here, is colored green. As a one-to-one order was not practical, some learning objectives of the Learning Objective Catalogue for Patient Safety were referenced with several learning objectives of the NCLM. An exact fit of the learning objective was indicated with an underlined NCLM-Learning goal number in the reference. Overall, seven of the 38 learning goals could not be referenced and 12 learning objectives are complementary to the existing learning objectives of the NCLM. This inexact fit is not unexpected as the Learning Objective Catalogue for Patient Safety was developed with the goal to integrate the topic into German undergraduate medical education on an internationally competitive basis. Due to lack of space, the reference to the NCLM is not part of this article but is available online as supplementary material (see attachment 2 ).

#### Additional information for handling the Learning Objective Catalogue for Patient Safety in Undergraduate Medical Education

Some additional information is presented complementarily to the Learning Objective Catalogue for Patient Safety in Undergraduate Medical Education. The interprofessional context will be incorporated in the implementation of the learning objectives. However, the Learning Objective Catalogue for Patient Safety in Undergraduate Medical Education was not developed for all health care professions. Instead, it was, against the background of our association, developed for the established undergraduate medical education programs. The Learning Objective Catalogue includes learning objectives from years of undergraduate medical education but not postgraduate specialty training. The educational realization and assignment to courses and specialties remains at the individual faculties and instructors. 

The learning objectives do not have to be taught chronologically. Rather, the order presented here serves as guidance, in which specialties and courses of a faculty’s learning objectives might have already been entirely or partly implemented. Additionally, the Learning Objective Catalogue for Patient Safety serves as an overview in which learning objectives of the *Basics* chapter have to be acquired before more advanced learning objectives can be acquired. The Learning Objective Catalogue for Patient Safety is developed in a way that learning objectives can be implemented longitudinally during undergraduate medical education. An intensive course or singular lesson for a few selected students does not appear sufficient to us.

The topics patient safety and error management are complex in nature and one individual cannot teach all learning objectives. The planning committees of curricula at the individual faculties should seek to obtain an expert’s assistance for these learning objectives in order to guarantee that medical students can actually achieve these learning objectives. 

## Discussion

The overall goal of the project was to develop fundamental recommendations for the implementation of structured patient safety curricula of medical students at medical faculties for the German speaking countries. The most relevant learning objectives for the topic patient safety that medical students should have achieved at the end of their studies should be defined. These recommendations were developed during two committee sessions during the annual meeting of the GMA and two multi-day workshops of the committee and review of the learning objectives through external professional experts. This resulted in the Learning Objective Catalogue for Patient Safety with three chapters and a hierarchical organization of the topics on three levels and 38 learning objectives overall.

In comparison to other learning objective catalogues for patient safety [5], [9], [10], it is noteworthy that the Learning Objective Catalogue for Patient Safety includes relatively few learning objectives. This might be an indication that the Learning Objective Catalogue for Patient Safety for Undergraduate Medical Education was developed with the explicit target of being in step with practice and manageable for curriculums planning at the medical faculties. Essentially, basic knowledge and skills of future physicians are represented here. This learning objective catalogue does not address the individual student who has a strong interest in patient safety. Furthermore, this learning objective catalogue is to be understood as a suggestion for a common ground for the implementation of structured curricula and is not claiming to be exhaustive. The learning objectives included in the Learning Objective Catalogue are the most important learning objectives for patient safety from the perspective of the committee. These learning objectives should be a substantial part of undergraduate medical education.

The freedom in how to implement the Learning Objective Catalogue for Patient Safety is deliberately left with the faculties. This has been done in light of the possible adaption strategies in the recently published NCLM.

The assignment of the type of learning objective (knowledge, attitudes, skills) was not distinct. It is without question that attitudes are difficult to teach and an assessment is possible but not trivial [12]. Change of attitudes through well-structured courses are often in contrast to inconsiderate medical practices and norms. Hence, despite all of the enthusiasm about the implementation of the topic into medical curricula, a quick change of unfavorable attitudes towards patient safety in everyday medial practice is not likely. The learning objectives described here target a compromise between eligible long-term development of the role of a physician and a safety culture and the short-term, practical implementation in medical faculties against the background of curricular and economic constraints.

The German and international evidence for the implementation of patient safety curricula and especially their effect on outcome parameter is sparse. For example, for physician-patient-communication, it is known that no direct relation between improved communication and healthcare provider parameters can be established [13]. Guidelines for the implementation of existing evidence is sparse as well [14]. For a lot of singular topics, it is unclear who - and with which qualification - is qualified to teach the learning objectives. 

Consequently, in the upcoming years, the aim is not only to establish structured and comprehensive patient safety education for all health care professionals, but also to critically and evaluate their effectiveness. Despite the fact that this emerging need for research is a challenge for all of those involved, it is an important next step to establish effective and sustainable structures for dealing with errors in medicine and create the best possible patient safety.

This publication is a manageable Learning Objective Catalogue and a product of collaboration from representatives of no less than 13 medical faculties. The intention is to advance the discussion of the topic in medicine within the German-speaking countries and the establishment of educational structures. It would be desirable that the Learning Objective Catalogue for Patient Safety in Undergraduate Medical Education serves as a foundation for constructive, professional deliberation with the topic at the individual faculties and initiates numerous curricular structures. 

## Notes

The position paper was accepted by the GMA executive board at 01-25-2016.

## Acknowledgements

The authors would like to thank the German Association for Medical Education for the financial support of the committee meetings in preparation of the manuscript. Furthermore, the authors are grateful to all experts for the critical feedback to individual learning objectives of the Learning Objective Catalogue for Patient Safety in Undergraduate Medical Education.

## Competing interests

The authors declare that they have no competing interests.

## Supplementary Material

Table 1: Learning Objective Catalogue for Patient Safety in Undergraduate Medical Education

Reference of NCLM and Learning Objective Catalogue for Patient Safety in Undergraduate Medical Education, Supplementary material (only available in German)
