# Supplementary material for: The Learning Objective Catalogue for Patient Safety in Undergraduate Medical Education – A Position Statement of the Committee for Patient Safety and Error Management of the German Association for Medical Education
Source: GMS J Med Educ. 2016 Feb 15;33(1):Doc10. doi: 10.3205/zma001009 (PMC4766934; doi:10.3205/zma001009)
Supplement: Table 1: Learning Objective Catalogue for Patient Safety in Undergraduate Medical Education [file JME-33-10-s-001.pdf]

No.	Level 1	Level 2	Level 3	Type (A, K, S)	Learning Objectives
**1. Basics 10-15%**					
1a	Terms and definitions		Error	K	The student knows the established definitions for the term "error" within the context of (clinical) medicine and is able to name the most important error types. The student can define the chain-of-error and a model that explains it (e. g. Swiss-cheese-model).
1b			Guilt and Responsibility	K	The student knows the difference between "guilt" and "responsibility" within the context of clinical practice and can name these differences.
1c	Law		Patients' rights and medical confidentiality	K	The student knows the patients' rights in its essential main features, as well as the preconditions and limitations of medical confidentiality and can illustrate its meaning for his/her action by means of concrete examples.
1d			Criminal-, Civil- and Vocational law	K	The student knows the differences between criminal, civil, and vocational law and can name the resulting different legal consequences. S/he can name possible legal consequences of medical errors.
1e			Transferal of negligence	A K	The student knows the term transferal of negligence; he/she can illustrate its meaning using an example from clinical practice and reflect his/her own clinical practice accordingly. He/she can differentiate between tasks that are delegable and tasks that are not delegable and name examples.
1f	Extent, Epidemiology	Hotspots	Hygiene	K S	The student knows frequent errors in hygiene and their effect on patient safety and can name these. Further he/she can reflect his/her own hygiene behavior within clinical everyday critically. The student knows that hygiene standards exist in each clinic.
1g			Safety of Pharmacotherapy	K S	The student knows about frequent errors in pharmacotherapy and their effect on patient safety and can name these. Further s/he can reflect behavior for the area of pharmacotherapy critically.
1h			Diagnoses-and diagnostic evaluation	K S	The student knows that errors can even happen in the area of diagnoses and diagnostic evaluation and is able to critically reflect an example accordingly.
1i			Wrong site/patient, remained foreign objects	K	The student knows about rare critical incidents, (e.g. patient-/site confusion or that foreign objects remain) which frequently have drastic consequences.
1j			Missing prophylaxis (falls, thrombosis, pulmonary embolism, decubitus)	K	The student knows about critical incidents (e.g. falls, thrombosis, decubiti) which result from a lack of prophylaxis.
1k		adverse events	Large scale studies	K	The student knows important, current "large-scale-studies" to errors in medicine, as well as the relevant results of these studies.

2. Recognize Causes as Foundation for Proactive Behavior 40%

2a	Complex System	Organizational factors	Interlinking (close, loose)		K	S	The student knows the system theory of incident genesis and can name differences to the person-centered approach. S/he is familiar with characteristics that promote emergence of error.
2b			Hierarchies		K		The student can illustrate the negative effect of a pronounced hierarchy on the patient safety.
2c		Patient factors	Polypharmacy		K		The student knows patient factors (e.g. polypharmacy and multi-morbidity) that contribute to the complexity of the system and promote emergence of error.
2d		Individual Factors	Limitations of human capability	A	K		The student knows that the human capability is limited and knows psychological and physical factors, which promote emergence of error.
2e			Evaluation of the own competence	A	K		The student knows that the own competence is limited and that an overestimation of one's own capabilities promotes the emergence of error.
2f		Interaction	Patient		K		The student knows that an insufficient informed consent regarding the treatment options with their advantages and disadvantages and risks and the option to do nothing constitutes an error.
2g	Interfaces		Team	A	K		The student knows that there are diverse types and characteristics of teams, which influence teamwork and which can promote emergence of error.
2h			Documentation	A	K		The student knows that an insufficient documentation promotes the emergence of error and can reflect examples accordingly.
2i			Hand-over	A	K		The student knows that interfaces in providing health-care can promote the emergence of error and can name examples accordingly. The student knows that an incomplete patient hand-over promotes the emergence of error.
2j			Human-Machine		K		The student knows that the interface human-machine promotes the emergence of error and can name examples accordingly.

**3. Approaches for Solutions 45-60%**							
3a	Strategies for optimization of patient-safety	Existing standards	Documents	A	K	S	The student knows existing standards to increase patient safety like i.e. guidelines (AWMF), checklists,<br>Recommendations for practice and clinic-specific standard operating procedures and has applied examples accordingly.
3b		Learning from Success/ Failure	CIRS, vigilance systems	A	K	S	The student knows CIRS (Critical Incident Reporting System) and its goals and as analyzed one exemplary report. The student knows vigilance systems, i.e. medical device and pharmacovigilance systems (FIDMD: Federal Institute for Drugs and Medical Devices).
3c			Morbidity- und Mortality-conferences		K		The student knows morbidity and mortality conferences and its goals.
3d			Root Cause Analysis		K		The student knows the method root cause analysis and can illustrate it.
3e			Patient identification	A	K	S	The student knows the principals of patient identification as an active process of perception, recognition and reassurance. The student knows diverse possibilities for patient identification and can apply these.
3f			Score systems	A	K	S	The student knows score systems and the goal to increase health care safety through its use. The student has applied a score using an example.
3g			Electronic / mobile tools		K		The student knows diverse electronic and mobile tools and their advantages and disadvantages regarding patient safety.
3h			High-safety		K	S	The student is familiar with the “high-safety organization” and can name strategies how these organizations achieve their high resilience towards critical incidents.
3i	Culture of Communication	Patient / Relative	Ppen disclosure	A	K	S	The student knows the model of complete open disclosure and can apply it towards patients as well as towards relatives.

3j		Informed consent	A	K	S	The student can provide a comprehensible informed consent including diverse treatment options with their advantages and disadvantages and risks and the option to do nothing.
3k		Handling of emotions		K		The student knows which emotions can occur in relation with critical incidents.
3l	Team	Second victim	A	K	S	The student knows that a critical incident can lead to acute stress reactions, which can be counteracted with appropriate countermeasures (resilience).
3m		Speak-up	A	K		The student knows that the expression of doubts on all hierarchical levels can contribute to the prevention of errors.
3n		Leadership competencies	A	K		The student knows the characteristics of leadership competency and the related actions and communicative tasks and their importance for patient safety.
3o		Supervision / Debriefing	A	K		The student knows the professional options for support in regarding critical incidents.
3p		Handover competencies		K	S	The student knows characteristics of a complete patient hand-over and can apply it using an example.
3q		Safe communication		K	S	The student knows diverse approaches of non-technical skills and can apply a technique using an example.

The type of the learning objective includes Attitudes (A), Knowledge (K), and Skills (S).
